# Spontaneous Thyroid Hemorrhage Caused by Langerhans Cell Histiocytosis: A Case Report and Literature Review

**DOI:** 10.3389/fendo.2021.610573

**Published:** 2021-05-19

**Authors:** Jingying Zhang, Chengchen Wang, Chuanshuai Lin, Binglong Bai, Mao Ye, Dapeng Xiang, Zhiyu Li

**Affiliations:** ^1^ Department of Thyroid Surgery, School of Medicine, Second Affiliated Hospital of Zhejiang University, Hangzhou, China; ^2^ Department of General Surgery, Hangzhou Mingzhou Hospital, Hangzhou, China

**Keywords:** Langerhans cell histiocytosis, thyroid, spontaneous thyroid hemorrhage, thyroidectomy, chemotherapy

## Abstract

**Purpose:**

Langerhans cell histiocytosis (LCH) is a rare clonal disorder of Langerhans antigen-presenting cells. However, thyroid LCH involvement is relatively rare. We present the first case of spontaneous thyroid hemorrhage due to LCH progression and discuss the clinical features, diagnosis, and treatments of thyroid LCH in a literature review.

**Methods:**

Clinical data were collected. Previously published articles on thyroid LCH involvement were reviewed to assess the clinical features, diagnosis, and treatments for thyroid LCH.

**Results:**

A 54-year-old female presented with a multi-system LCH, affecting the uterus, liver, pituitary gland, and thyroid gland. Clinical stability was achieved after systemic chemotherapy. After 7 years of regular follow up, the patient complained of a sudden painful neck swelling and progressive dyspnea. Computed Tomography revealed bilateral goiter with hematoma, and the patient was diagnosed with spontaneous thyroid bleeding based on her clinical symptoms and radiological findings. The patient was incubated to relieve airway compromise and partial thyroidectomy was performed for definitive treatment. Pathological evaluation further confirmed the diagnosis of thyroid LCH. The patient recovered well after surgery.

**Conclusion:**

Spontaneous thyroid bleeding due to thyroid LCH progression is extremely rare. Treatments for LCH vary depending on the severity of the disease. We suggest that, for patients with multi-system LCH with thyroid lesion, long-term active surveillance of thyroid hormone concentrations, and thyroid gland volume is required. Physicians should be alert of the potentially life-threatening spontaneous thyroid hemorrhage when aggravated diffuse goiter and hypothyroidism appear. Further investigation is required to establish the guidelines for thyroid LCH treatment.

## Introduction

Langerhans cell histiocytosis (LCH) is a rare disease caused by Langerhans cell clonal hyperplasia (1). It can involve several organs and tissues. Including the bones, the lungs, the skin, the liver, the lymph nodes, and the pituitary gland. Thyroid involvement is relatively rare and most commonly occurs as part of multi-system LCH (2). Clinical manifestations of LCH depend on the site of involvement. Common symptoms of bone LCH included painful masses and osteolytic lesions. LCH may cause diabetes insipidus when the pituitary gland is involved. Conversely, LCH with thyroid involvement mostly manifests as diffuse or nodular enlargement with or without hypothyroidism. Neck pain, dysphonia, and obstructive symptoms are rare (2). Although it is highly vascularized, spontaneous hemorrhage of the thyroid gland is rare and may cause lethal acute airway obstruction. In this article, we report the first case of a patient with spontaneous thyroid hemorrhage due to LCH involvement and further discuss the clinical features, diagnosis, and treatment for thyroid LCH in a literature review.

## Case Description

### Patient History

**Figure 1:**
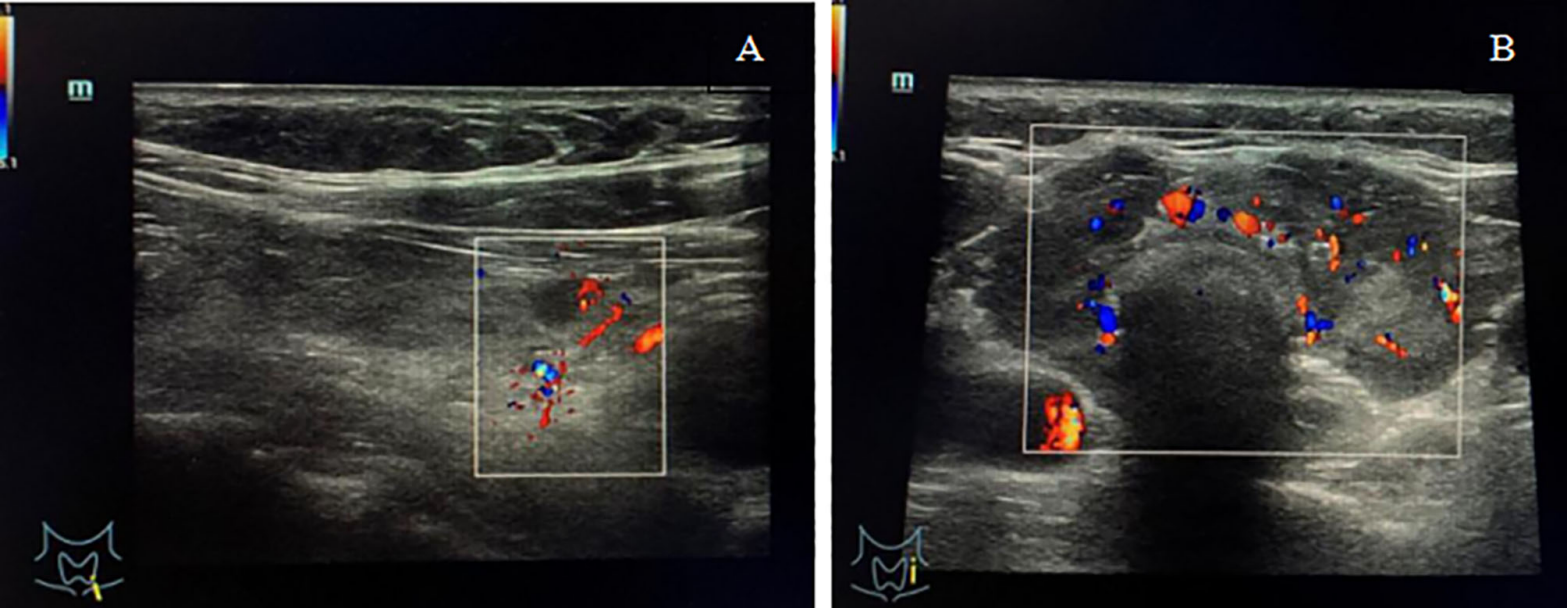
**(A)** Ultrasonography showed bilateral thyroid reduction and multiple nodules (<0.5 cm in diameter) in the left lobe with normal blood flow signals. **(B)** Ultrasonography showed thyroid enlargement with diffuse reduction in gland echo and abundant blood flow signals.

A 54-year-old female patient presented to the local hospital with a sudden painful neck swelling and progressive dyspnea. A medical history revealed that the patient underwent an uterectomy due to a uterine mass, which was suspected of malignancy by biopsy, and LCH was diagnosed by postoperative pathology 7 years prior. A systemic inspection showed suspicious lesions in the thyroid gland, liver, and the pituitary gland. Thyroid fine-needle aspiration (FNA) biopsy was performed, and a pathologic examination further confirmed LCH. Therefore, the patient was clinically diagnosed with multi-system LCH involving “risk organs”. The patient underwent several rounds of systemic chemotherapy, including vincristine (150 mg d1^−3^) and prednisone (1000 mg d1^−3^, q4w, 3 times); etoposide (150 mg d1^−3^) and prednisone (1000 mg d1^−3^, 1 time); and cytarabine (180 mg d1^−5^, q4w, 10 times), and she achieved clinical remission. A thyroid function test showed slight hypothyroidism, and thyroid ultrasonography revealed bilateral thyroid reduction with multiple nodules (<0.5 cm in diameter) in the left lobe (**Figure 1A**). The patient was then prescribed levothyroxine (50 µg/day) and desmopressin acetate (0.075 mg/day) for hypothyroidism and diabetes insipidus. After 1 year of follow up, magnetic resonance imaging (MRI) showed hypothalamic pituitary enlargement and increased number of liver nodules. Ultrasonography revealed that the thyroid gland was significantly larger than before (**Figure 1B**). The patient was given another dose of cytarabine chemotherapy180 mg d1^−5^ and achieved partial remission. In spite of regular follow up, the patient suddenly presented with a painful neck swelling and progressive dyspnea and denied any form of trauma. The patient had received tracheal intubation at the local hospital before being referred to our hospital.

An initial examination revealed an obvious swelling in the anterior cervical region, which was tender to touch. A thyroid function test at admission reported severe hypothyroidism with a thyroid-stimulating hormone concentration of 8.45 mIU/L (normal range: 0.35–4.94 mIU/L), a free thyroxine concentration of 6.46 pmol/L (normal range: 9.01–19.05 pmol/L), a total thyroxine of 45.28 nmol/L (normal range: 58.1–140.6 nmol/L), a free triiodothyronine concentration of 2.38 pmol/L (normal range: 2.63–5.70 pmol/L), a total triiodothyronine concentration of 0.82 pmol/L (normal range: 0.89–2.44 pmol/L). Neck computed tomography revealed bilateral diffuse goiter with hematoma. Contrast-enhanced computed tomography was rejected because of iodine allergy. A diagnosis of spontaneous thyroid hemorrhage was made based on the clinical symptoms and radiological findings.

## Results

### Case Treatments and Outcome

**Figure 2:**
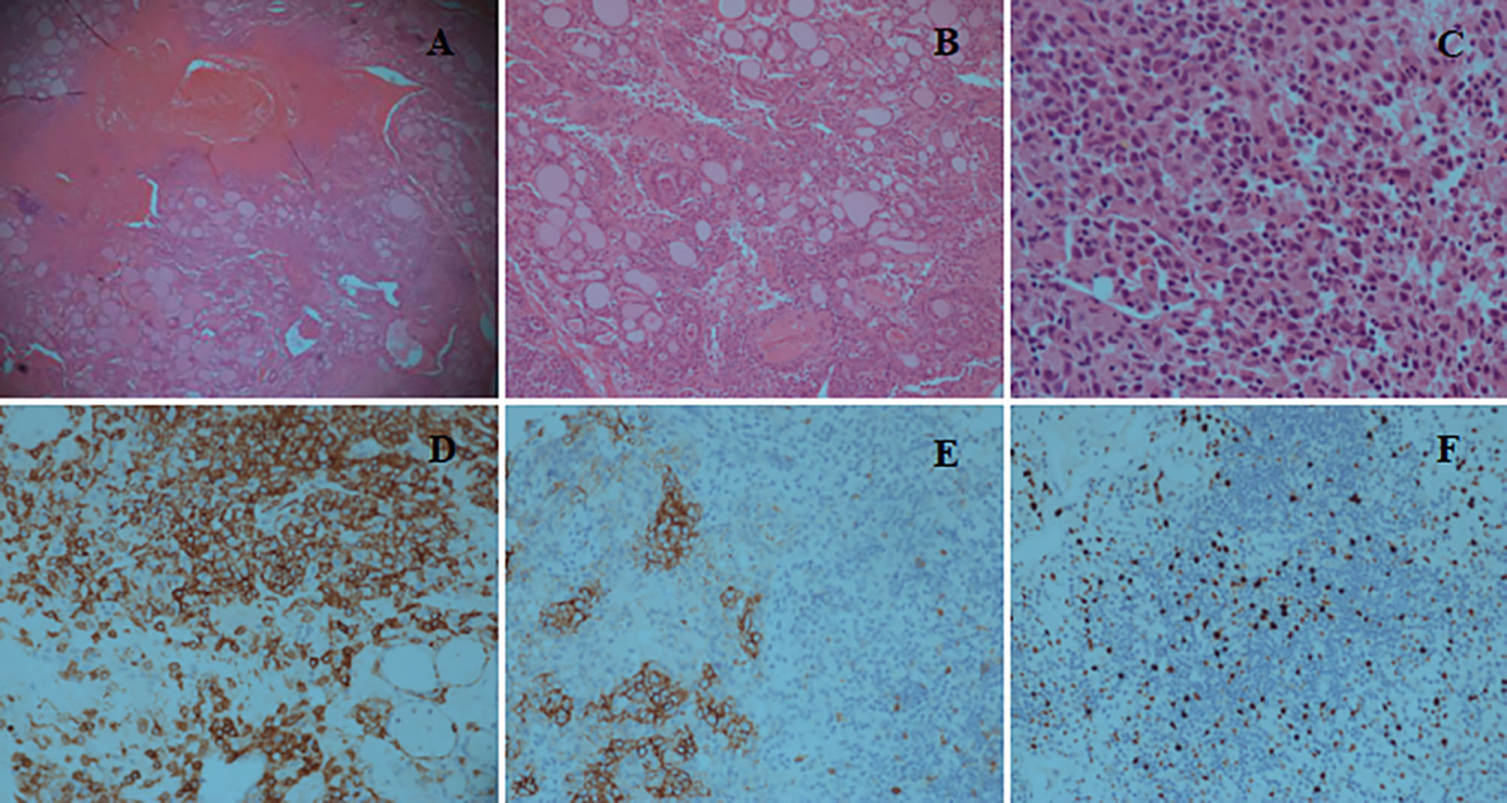
**(A)** Hematoxylin and Eosin (H&E) staining (×5) reveals tissue necrosis and the infiltration composed of Langerhans cells, lymphocytes, and eosinophils. **(B)** H&E staining (×10) shows sheets and islands of nuclear atypia cells infiltration between the thyroid follicles. **(C)** H&E staining (×40) reveals diffuse proliferation of nuclear atypia cells with typical nuclear grooves and indentation. **(D)** Immunochemical staining (×20) shows that Langerhans cells are strongly positive for CD1a, whereas thyroid follicular epithelial cells are negative. **(E)** Immunochemical staining (×20) shows positive reaction for CD56. **(F)** Immunochemical staining (×20) shows slightly positive reaction for Ki-67.

The patient was transferred to the intensive care unit. During surgery, we observed massive blood clots causing surrounding tissues edema, while the thyroid gland was severely diminished and only partial thyroid tissue remained, which was fragile and easily bled upon touching, especially the thyroid isthmus. No active bleeding was found at that time. Thyroid isthmusectomy was performed, and the hematoma was removed. Prophylactic tracheotomy was performed in case of secondary postoperative bleeding and dyspnea. A pathological examination showed a classic phenotype with sheets and islands of nuclear atypical cells infiltration, with strongly positive immunochemical staining for CD1a, which confirmed the diagnosis of LCH. Meanwhile, immunochemical staining showed positive staining for CD56 and Ki-67 (**Figure 2**). The patient was successfully discharged from hospital 1 week after surgery. After 10 months, the tracheostomy cannula was removed, and the incision was healing well. Currently, the patient is under close follow-up.

### Thyroid LCH Involvement

The PubMed, Web of Science and Medline databases were searched for articles published between January 1, 2010 and April 1, 2020, using the keywords: “thyroid” and “Langerhans cell histiocytosis”, “Histiocytosis X”, “Eosinophilic Granuloma”, “Hand-Schüller-Christian disease”, “Letterer-Siwe disease”. And the references of each article were searched for further relevant publications. A total of 49 relevant articles were published between 2010 and 2020, but 22 articles were excluded due to incomplete information about the clinical feature and treatment of thyroid LCH. Therefore, in 27 articles, 29 cases of thyroid LCH involvement were reviewed (**Table 1**) (2–28). LCH involving the thyroid gland is more common in adults compared with children (25 adults *vs.* 4 children). Adult females are slightly more commonly affected than adult males (ratio of 1.27:1) (**Table 2**). Solitary thyroid involvement of LCH (24.14%) is rare and most cases (75.86%) present as part of multi-system LCH (**Table 2**).

**Table 1:** The cases of thyroid LCH involvement (in chronological order).

Gender	Age (years)	Gland feature	Thyroid function	Other thyroid disease	FNA	Other organ involvement	Treatment	Outcome/Reference
M	3	Goiter	N	NR	LCH*	lungs*	chemotherapy	no tumor recurrence after 1 year (3)
F	38	Nodules	Hypothyroidism	HT	Benign	lungs, liver	TT*	Discontinued (4)
M	23	Goiter	Subclinical hypothyroidism	NR	TC	pituitary, lymph nodes, lung and mandible	TT*, chemotherapy	Good disease control but no remission (4)
M	29	Goiter	NR	PTC	PTC, LCH*	bone (mandible), lung, skin and hypothalamo-pituitary	chemotherapy	trachea compression,death (5)
F	5	Goiter	Hypothyroidism	NR	LCH*	hypothalamo-pituitary	chemotherapy	died of unknown cause (6)
F	52	Nodules	N	NR	Benign	N	right hemithyroidectomy*	no evidence of systemic disease after 1 year (7)
M	37	Goiter	N	N	LCH*	spine*	spinal operation	no recurrence (8)
F	44	Goiter	Hypothyroidism	NR	MTC	N	left hemithyroidectomy*	disease-free at last follow-up 9 months later (2)
F	38	Nodules	N	N	LCH*	N	subtotal thyroidectomy*	no evidence of systemic disease after 3 months (9)
M	27	Goiter	N	N	LCH*	N	subtotal thyroidectomy*	no evidence of systemic disease after 6 months (9)
M	37	Goiter	NR	PTC	Unclear	lymph nodes*	TT*	died due to cardiac arrest with respiratory (10)
M	22	Goiter	Hypothyroidism	N	LCH*	hard palate*	chemotherapy	clinical complete remission (11)
F	35	Goiter	N	NR	LCH	pituitary*	chemotherapy	no recurrence of disease till present (12)
F	54	Goiter	Hypothyroidism	NR	LCH*	hypothalamo-pituitary	chemotherapy	NR (13)
M	45	Goiter	Subclinical hypothyroidism	NR	NR	N	TT*	now on regular follow-up (14)
F	26	Nodules	Hyperthyroidism	hyperthyroidism	NR	N	TT*	NR (15)
M	8	Goiter	N	N	PTC	lymph nodes	Thyroid biopsy*, chemotherapy	no evidence of systemic disease after 1 year (16)
M	27	Nodules	N	N	LCH*	the vertebral body of S1–2*	chemotherapy,ABMSCT	no tumor recurrence (17)
F	73	Nodule	Hypothyroidism	HT	PTC	N	TT*	NR (18)
F	39	Nodule	Hypothyroidism	N	Unclear	pituitary	surgery*	no evidence of systemic disease after 1 year (19)
F	22	NR	NR	PTC, HT	NR	pituitary and skin*	TT*, I-131 and high dose prednisone	transient improvement in TPS and skin, with no change in pituitary function (20)
F	27	Goiter	Secondary hypothyroidism	PTC	PTC	hypothalamo-pituitary and cervical lymph nodes	TT*, I-131 and chemotherapy	NR (21)
M	36	Nodule	N	N	LCH	perianal*	TT*, perianal operation and local radiotherapy	no recurrence after 6 months (22)
M	40	Goiter	NR	PTC	PTC	saddle area, lungs, palate*, and cervical lymph nodes	right hemithyroidectomy*, chemotherapy	no significant sign of recurrence after 2 years (23)
M	41	Nodule	NR	NR	NR	perianal and liver*	right thyroid resection*, radiotherapy, interleukin-2, chemotherapy	clinical remission after 5 years (24)
F	45	Goiter	NR	PTC	NR	lungs, bones* and lymph nodes	TT*, I-131 and chemotherapy	NR (25)
F	36	Goiter	N	PTC	PTC	skin* and cervical lymph node*	debulking thyroidectomy*, chemotherapy	stable clinical condition after 12 months (26)
F	3	Goiter	Hypothyroidism	NR	LCH*	lungs*	chemotherapy,resection of the bullae	no recurrence after 12 months (27)
F	37	Nodule	N	NR	NR	perianal and bones	TT*,chemotherapy	NR (28)

M, male; F, female; N, normal, NR, no report; HT, Hashimoto’s thyroiditis; TC, thyroid carcinoma; PTC, papillary thyroid carcinoma; MTC, medullary thyroid carcinoma; TT, total thyroidectomy; *Pathologically confirmed LCH.

**Table 2:** The demography, clinicopathologic features, diagnose, treatment and outcome of patients with thyroid LCH involvement.

Features		Ratio
Patient(M:F)		29(1:1.23)
	Child(M:F)	4(1:1)
	Adult(M:F)	25(1:1.27)
Thyroid gland		
	Goiter	62.07%(18/29)
	Nodule(s)	37.93%(11/29)
Thyroid function		
	N	37.93%(11/29)
	Hypothyroidism	31.03%(9/29)
	Hyperthyroidism	3.45%(1/29)
	Subclinical hypothyroidism	6.9%(2/29)
	NR	20.69%(6/29)
Other disease		
	HT	10.34%(3/29)
	PTC	24.14%(7/29)
	PTC with HT	3.45%(1/29)
	Hyperthyroidism	3.45%(1/29)
	N	27.59%(8/29)
	NR	37.93%(11/29)
FNA		
	LCH	37.93%(11/29)
	TC	27.59%(8/29)
	Benign	6.9%(2/29)
	Undetermined significance	6.9%(2/29)
	NR	20.69%(6/29)
Other involvement		
	N	24.14%(7/29)
	Multisystem	75.86%(22/29)
Treatment		
	Surgery of thyroid	34.48%(10/29)
	Chemotherapy	31.03%(9/29)
	Surgery+chemotherapy/radiotherapy	31.03%(9/29)
	Other	3.45%(1/29)
Treatment in adults		
	Surgery of thyroid	40%(10/25)
	Chemotherapy	20%(5/25)
	Surgery+chemotherapy/radiotherapy	36%(9/25)
	Other	4%(1/25)
Treatment in children		
	Chemotherapy	100%(4/4)
Outcome		
	No recurrence	58.62%(17/29)
	Alive with tumor	6.9%(2/29)
	Death from tumor	3.45%(1/29)
	Death from other reason	6.9%(2/29)
	NR	24.14%(7/29)

### Clinical Features

Most cases of LCH involving the thyroid gland presented with goiter or diffuse thyroid enlargement (62.07%). Almost one-third of cases present with a single thyroid nodule or multiple thyroid nodules (37.93%). Fewer than half of cases occur in patients with euthyroidism (37.93%), and one-third of cases occur in patients with hypothyroidism (31.03%). Subclinical hypothyroidism (6.9%) and hyperthyroidism (3.45%) are uncommon (**Table 2**). These presentations are nonspecific and can be easily confused with benign goiter or thyroid carcinoma.

### Diagnosis

Thyroid FNA is useful to establish the primary diagnosis. However, the results of FNA are often variable, showing benign (6.9%), papillary or medullary thyroid carcinoma (27.59%), or atypical of undermined significance (10.34%) (**Table 2**). The golden standard for diagnosis of LCH is electronic microscopy (Birbeck granules) or immunohistochemical staining (S100 and CD1a positivity) (2). Coexistence of thyroid LCH involvement with other thyroid diseases, such as Hashimoto’s thyroiditis (10.34%) and papillary thyroid carcinoma (24.14%), is not uncommon (**Table 2**).

### Treatment

Of 29 patients, 10 (34.48%) underwent surgical resection for thyroid involvement, 9 (31.03%) underwent chemotherapy only, and 9 (31.03%) underwent surgery and chemotherapy or radiotherapy. Seven patients (24.14%) with solitary thyroid gland involvement with LCH underwent surgery only (**Table 2**).

### Outcome

More than half of the patients (58.62%) were followed up for 6 months to 5 years, and no evidence of disease recurrence was found. Two patients (6.9%) were alive but did not achieve remission. Only 1 patient (3.45%) died of disease recurrence (acute respiratory failure caused by the trachea compression and infiltration), whereas 2 patients (6.9%) died of other causes. In addition, for the remaining cases (24.14%), no follow-up outcome was reported (**Table 2**).

## Discussion

Langerhans cells are antigen-presenting cells resident in the epidermis, mucosae, or bronchial epithelium. LCH is a disease caused by the monoclonal proliferation of Langerhans cells. The annual incidence of LCH is 5 to 9/10^6^ in children younger than 15 years of age and 1 to 2/10^6^ in individuals older than 15 years of ages (29–31). The bones, skin, pituitary gland, liver, spleen, hematopoietic system, and lungs are most frequently affected by LCH. Thyroid LCH involvement is not frequent, affecting 9%-25% of individuals with LCH (32–35). To our knowledge, this is the first reported case of spontaneous hemorrhage caused by thyroid gland LCH in the literature.

For thyroid LCH, the characteristic clinical feature is diffuse goiter or a painless thyroid mass. Laboratory tests of thyroid function in patients with LCH may be variable. With thyroid ultrasonography, LCH involvement can be manifested as solid hypoechoic nodules or diffuse reduction of gland echo, which can be easily misdiagnosed as multinodular goiter, Hashimoto’s thyroiditis, thyroid cancer or lymphoma (9). In this case report, the patient presented with diffuse goiter and hypothyroidism at disease onset and was prescribed with oral levothyroxine to treat hypothyroidism. Thyroid hormone concentrations were maintained almost within the normal range (only free thyroxine was slightly lower at 8.90 pmol/L). However, ultrasonography showed an increased in thyroid gland volume and laboratory tests showed a lower thyroid hormone concentration at follow up, which may indicate LCH progression. The patient may have suffered primary hypothyroidism (LCH thyroid involvement) combined with secondary hypothyroidism (LCH pituitary involvement), since the thyroid-stimulating hormone concentration was not high enough, while the thyroid hormone concentrations were relatively low at first visit. Thyroid-stimulating hormone deficiency commonly develops in the context of hypopituitarism (33).

FNA might be helpful to diagnose of thyroid LCH (2). However, histology may be more sensitive as a diagnostic modality. To identify thyroid LCH, infiltration of lymphocytes and eosinophils with large cytoplasm should be observed in the thyroid gland. LCH should be confirmed the presence of Birbeck granules using electron microscopy or immunohistochemical positivity for S100 and CD1a (1, 2).

There are available clinical guidelines for the treatment of pediatric LCH (34). However, there are no well-accepted treatments for adult LCH because of limited case studies and series (1, 36–38). Many clinical trials indicated that different lesion location and risk stratification can determine LCH treatment (24, 38). The liver, spleen and hematopoietic system are risk organs, which subdivide systemic LCH into high-risk and low-risk groups. The majority of cases of multi-system LCH involve at least one risk organ and indicate poor outcomes. For single-system thyroid LCH, partial resection should be used for treatment, with or without radiotherapy or mild chemotherapy (39). For multi-system LCH, systematic chemotherapy is recommended. Low-risk LCH should adopt low-dose and low-frequency chemotherapy to prevent diabetes insipidus or other late complications. Vinblastine combined with prednisone is the traditional chemotherapy regimen for pediatric LCH (40), while cytarabine might be better for adult LCH (36–38). On the contrary, high-risk LCH should adopt prolonged and intensive combination chemotherapy based on cladribine, cytarabine, 6-mercaptopurine, and methotrexate to improve long-term survival (41, 42). Recent research shows that mutated *BRAF* is presented in the majority of LCH cases and increases the risk of recurrence, suggesting that vemurafenib, which is an inhibitor of the B-Raf enzyme, can be used to treat LCH (1, 43, 44). An open-label nonrandomized study of 26 patients with *BRAF* V600–mutant LCH and Erdheim-Chester disease indicated that vemurafenib had prolonged efficacy, with a 62% confirmed overall response rate (45). Consistently, Donadieu et al. demonstrated that vemurafenib seem to be effective in children with refractory BRAFV600E-positive LCH (46). Prospective clinical trials are needed to determine the treatment duration for LCH patients.

However, definitive treatment for thyroid LCH is still controversial due to the lack of prospective randomized studies. Solitary thyroid LCH involvement is difficult to distinguish from other thyroid diseases, especially when it manifests as large goiter or painless nodules. Seven patients with solitary thyroid LCH involvement underwent surgery only, including hemithyroidectomy, subtotal thyroidectomy, or total thyroidectomy. In most of these cases (5 reported cases), no evidence of systemic disease or recurrence was noted (**Table 1**). There is no evidence to prove that adjuvant chemotherapy or radiotherapy after surgical resection improve the outcomes of primary thyroid LCH (2, 5, 7). Once thyroid LCH is diagnosed, it is necessary to perform systemic inspections, such as lung computed tomography, bone scintigraphy, liver ultrasonography, or positron emission tomography–computed tomography to definitively diagnose multi-system LCH, which requires systemic therapy (17, 22). Chemotherapy regimens, such as vinblastine, etoposide, prednisone, and cytarabine have been used to treat aggressive disease. In our report, the patient was diagnosed with multi-system LCH affecting the uterus, thyroid gland, liver, and pituitary gland. The patient underwent systemic chemotherapy with different regimens, including vinblastine/prednisone, etoposide/prednisone, and cytarabine, and finally achieved clinical remission. After regular follow up, LCH recurrence occurred with thyroid gland enlargement and aggravated hypothyroidism, which finally caused spontaneous thyroid hemorrhage and dyspnea. Thus, close long-term follow up seems crucial.

Spontaneous thyroid hemorrhage is uncommon. Most cases occur following direct neck trauma or FNA; they rarely follow indirect trauma near the neck and an excessive Valsalva maneuvers (47–50). Aggressive goiter or malignant tumor can increase and alter thyroid gland vascularity (51–53). The risk of bleeding is increased due to immature angiogenesis, arteriovenous shunt, increased vascular flow, and thyroid gland fragility. Anticoagulants can also increase the severity of spontaneous bleeding (54). Elbers et al. (55) indicated that low thyroid hormone concentrations could shift the hemostatic system towards hypocoagulability and a hyperfibrinolytic state, which may increase the bleeding risk. The pathogenesis of thyroid hemorrhage in this case is hard to determined. It may have been associated with aggravated hypothyroidism and progressive diffuse goiter due to disease recurrence. Therefore, for patients with thyroid LCH, active surveillance of thyroid hormone concentrations and thyroid gland volume is required, and a sufficient dose of levothyroxine is needed for patients with hypothyroidism. Spontaneous thyroid hemorrhage should be alerted when the treatment response is poor or when patients present with severe thyroid enlargement and hypothyroidism, which can cause obstructive symptoms even death.

## Conclusion

Thyroid LCH is a rare disease. Spontaneous thyroid bleeding caused by thyroid LCH was first reported in this article. FNA and incision biopsy have a certain diagnostic value. Multi-system LCH should be evaluated by systemic examinations. Treatment options vary depending on the extent of disease. For thyroid LCH, long-term active surveillance of thyroid hormone concentrations and thyroid gland volume is required. Clinicians should be alert of spontaneous thyroid bleeding, especially for patients with aggravated diffuse goiter and hypothyroidism. Further studies on treatments and surveillance for thyroid LCH are urgently needed.

## Data Availability Statement

The original contributions presented in the study are included in the article/supplementary material. Further inquiries can be directed to the corresponding author.

## Ethics Statement

Written informed consent was obtained from the individual(s) for the publication of any potentially identifiable images or data included in this article.

## Author Contributions

JZ, CW, and ZL contributed to the study conception and design. CW, JZ, and CL searched prior articles and finished data analysis. BB, MY, and DX collected the clinical data. JZ and CW wrote the first draft of the manuscript. JZ revised the manuscript. All authors contributed to the article and approved the submitted version.

## Funding

This work was supported by the National Natural Science Foundation of China (81802334).

## Conflict of Interest

The authors declare that the research was conducted in the absence of any commercial or financial relationships that could be construed as a potential conflict of interest.
